# Effect of Structured Exercise on Blood Pressure in Patients With Rheumatoid Arthritis: A Systematic Review

**DOI:** 10.7759/cureus.98712

**Published:** 2025-12-08

**Authors:** Maryam Walizada, Gowtham Siddi, Srirachana Reddy Gumireddy, Mariette Anto, Israa Elkashif, Sindhu Vithayathil, Summayya Anwar

**Affiliations:** 1 Internal Medicine, Mary Washington Hospital, Fredericksburg, USA; 2 Internal Medicine, NRI Academy of Medical Sciences, Guntur, IND; 3 Medicine, Apollo Institute of Medical Sciences and Research, Hyderabad, IND; 4 Orthopaedics, King's Mill Hospital, Sutton-in-Ashfield, GBR; 5 Psychiatry, Royal College of Surgeons in Ireland, Dublin, IRL; 6 General Medicine, American University of Antigua, Coolidge, ATG; 7 Biosciences, COMSATS University Islamabad, Islamabad, PAK; 8 Research and Development, California Institute of Behavioral Neurosciences & Psychology, Fairfield, USA

**Keywords:** blood pressure, cardiovascular risk factor, physical activity, rheumatoid arthritis, structured exercise

## Abstract

Rheumatoid arthritis (RA) is a chronic inflammatory autoimmune disease that primarily affects joints and causes mobility problems. RA is associated with high mortality and morbidity as a result of the increased prevalence of cardiovascular disease (CVD) and cardiovascular (CV) risk factors, such as blood pressure (BP), in these patients. Exercise has a beneficial effect on CVD and CV risk factors and improves the overall well-being of RA patients. This systematic review aims to investigate the effect of exercise on CVD and CV risk factors, specifically BP, in RA patients. We searched the relevant literature across databases such as PubMed, PubMed Central, Cochrane Library, ScienceDirect, and Google Scholar. In this study, we included English-language studies with full text and related information on the impact of exercise on CVD in RA patients. The study follows the Preferred Reporting Items for Systematic Reviews and Meta-Analyses (PRISMA) 2020 guidelines. We used the Joanna Briggs Institute (JBI) appraisal method for evaluating non-randomized clinical trials (RCTs), pilot studies, and observational studies, the Assessment of Multiple Systematic Reviews (AMSTAR) checklist for systematic reviews, and the Narrative Review Checklist for the narrative review studies. Eight relevant studies were included in this systematic review. The study shows that different types of exercise, from low to high intensity, have a beneficial effect on CVD and CV risk factors such as BP in RA patients. Exercise improves mortality, morbidity, and overall quality of life in this patient population. The study strongly recommends incorporating physical activity into the management of RA patients.

## Introduction and background

Rheumatoid arthritis (RA) is an autoimmune, chronic, inflammatory disease that affects approximately 0.5-1% of the general population [1]. It primarily affects synovial joints and causes joint deformity [2]. Patients with RA present with joint manifestations such as articular and periarticular swelling, pain, and stiffness, which may result in significant limitation in mobility. The prominent long-term consequences in the musculoskeletal system are inflammation-induced joint damage that predominantly affects functional capacity and daily activities. In addition, patients suffer from fatigue, depression, and anxiety [1,3]. In RA, the body produces antibodies against itself. These antibodies are called autoantibodies, such as rheumatoid factor (RF) and anti-citrullinated protein antibody (ACPA), and these are produced mainly by the lungs. RA has a preclinical phase, without clinical symptoms, and this phase can last for years before the clinical onset of disease. The most critical risk factor of RA is located within the class II major histocompatibility (MHC) locus. A total of 70% of RA patients have positive human leukocyte antigen (HLA)-DR4 compared to 30% of patients without RA [4]. These figures indicate that HLA-DR4 is an essential genetic susceptibility factor for RA, as individuals carrying this allele have a substantially higher risk of developing the disease than the general population. However, its presence alone is not diagnostic.

Structured exercise has multiple beneficial effects in the general population and patients with chronic diseases, including decreasing the risk of coronary artery disease, stroke, diabetes, and hypertension [5]. In RA patients, physical activity is beneficial in improving function and reversing cachexia without increasing disease severity, and it is highly possible to decrease cardiovascular (CV) risk [6].

High blood pressure is often associated with RA and causes the development of cardiovascular disease (CVD) in these patients [7,8]. However, limited data are available on factors influencing blood pressure in this patient population [7]. Patients with a low level of disease activity score have lower blood pressure [9]. Regular exercise has been shown to reduce blood pressure in patients with RA [7], and treatment with infliximab, a tumor necrosis factor-α inhibitor, is also associated with decreased blood pressure in this population [9], suggesting that both non-pharmacologic and biologic therapies that lower disease activity may contribute to improved blood pressure control.

The management of RA needs coordination of many medical specialties, including general practitioners, rheumatologists, cardiologists, exercise physiologists, and psychologists, to achieve optimal outcomes, including better disease control, improved functional status and quality of life, and reduced complications, morbidity, and mortality [10].

The increased CV morbidity and mortality seen in RA patients is independent of CV risk factors such as hypertension [1,10]; the cumulative inflammatory effect of RA and antirheumatic medication-related cardiotoxicity seems to be an important contributor [2,3]. In addition, other reasons can be the higher prevalence and worse outcome of ischemic heart disease in this patient population [1,10]. Another contributor to CVD in RA patients is low cardio-respiratory fitness (CRF), but exercise has shown a beneficial effect on it [6]. While structured exercise has been shown to improve CVD and decrease CV risk factors, including blood pressure [7,8], evidence specifically addressing its impact on blood pressure in RA patients is limited [9].

Aims and objectives

This systematic review aims to evaluate the effect of structured exercise on blood pressure in RA patients. The specific objectives are to (1) summarize the evidence on changes in systolic and diastolic blood pressure following structured exercise interventions in adults with RA, and (2) describe the main characteristics of these exercise programs, including type, frequency, intensity, and duration.

## Review

Methodology

This systematic review was implemented according to the Preferred Reporting Items for Systematic Reviews and Meta-Analyses (PRISMA) 2020 guidelines [11,12].

Databases and Search Strategy

We carried out a systematic search across five databases: PubMed, PubMed Central (PMC), Cochrane, Google Scholar, and ScienceDirect. All databases were last searched on July 7, 2025. The search fields were determined according to the keywords reported in the previous studies and through Medical Subject Headings (MeSH). Details are presented in Table 1.

**Table 1:** Included studies using each database.

Database	Keywords	Search strategy	Search results (before filter)	Filters	Search results (after filter)
PubMed	structured exercise, blood pressure, rheumatoid arthritis	(("Exercise/physiology"[Majr]) OR "Work/physiology"[Majr] OR "Movement/physiology"[Majr]) AND ("Blood Pressure/physiology"[Majr] OR "Hypertension/physiopathology"[Majr] OR "Arterial Pressure/physiology"[Majr]) AND ("Arthritis, Rheumatoid/rehabilitation"[Majr] OR "Arthralgia/rehabilitation"[Majr])	468	Free full text, English	138
Google Scholar	structured exercise, blood pressure, rheumatoid arthritis	"Structured exercise" AND "blood pressure" AND "rheumatoid arthritis"	950	Free full text, English	951
PubMed Central (PMC)	structured exercise, blood pressure, rheumatoid arthritis	"Structured exercise"[All Fields] AND "blood pressure"[All Fields] AND "rheumatoid arthritis"[All Fields]	209	Filters not available; will be applied manually during screening	209
ScienceDirect	structured exercise, blood pressure, rheumatoid arthritis	"Structured exercise" AND "blood pressure" AND "rheumatoid arthritis"	88	Open access; language filters not available and will be applied manually during screening	32
Cochrane Library	structured exercise, blood pressure, rheumatoid arthritis	("Structured exercise" OR "Supervised exercise") AND ("Hypertension" OR "Blood pressure") AND "Rheumatoid arthritis"	18	Free full text and English filters not available; will be applied manually during screening	18

Eligibility Criteria

The papers were chosen according to the Population, Intervention, and Outcome (PIO) items. The population included RA patients; the intervention was structured exercise; and the outcome was blood pressure. Additionally, we applied further inclusion and exclusion criteria. Inclusion criteria included articles in the English language, free full-text papers, randomized clinical trials (RCTs), observational studies, systematic reviews and meta-analyses, case reports, case studies, editorials, and papers focusing on all age groups. Exclusion criteria included patients not having RA, studies not assessing exercise intervention, studies not peer reviewed, studies not reporting cardiovascular outcomes like blood pressure, and grey literature.

Selection Process

All the relevant studies were collected in EndNote (Clarivate, Philadelphia, PA), and the replicated ones were excluded. We screened every single study by reading its title and abstract. After determining the suitable studies, we evaluated the included articles for the availability of the full text. Selected articles were reviewed based on inclusion and exclusion criteria. The targeted studies that met the requirements were included in this study.

Quality Appraisal

Appropriate quality assessment tools were applied to the selected papers based on their study design. We utilized the Joanna Briggs Institute (JBI) quality assessment tools to assess the non-randomized clinical trials, observational studies, and the pilot study [13]. Narrative review papers were evaluated using the Academy of Nutrition and Dietetics Narrative Review Checklist [14], and the single systematic review was assessed with the Assessment of Multiple Systematic Reviews (AMSTAR) checklist [15]. Two co-authors independently evaluated the quality of all included articles, and any disagreements were resolved by discussion.

Data Extraction

After selecting the eligible studies, two co-authors independently extracted data for this review. Extracted items included study design, type and intensity of exercise, duration of the intervention, and cardiovascular or blood pressure outcomes in patients with RA. This review focuses on the impact of exercise on blood pressure in RA patients, and all extracted data were checked by both co-authors before use in the review.

Results

**Figure 1:**
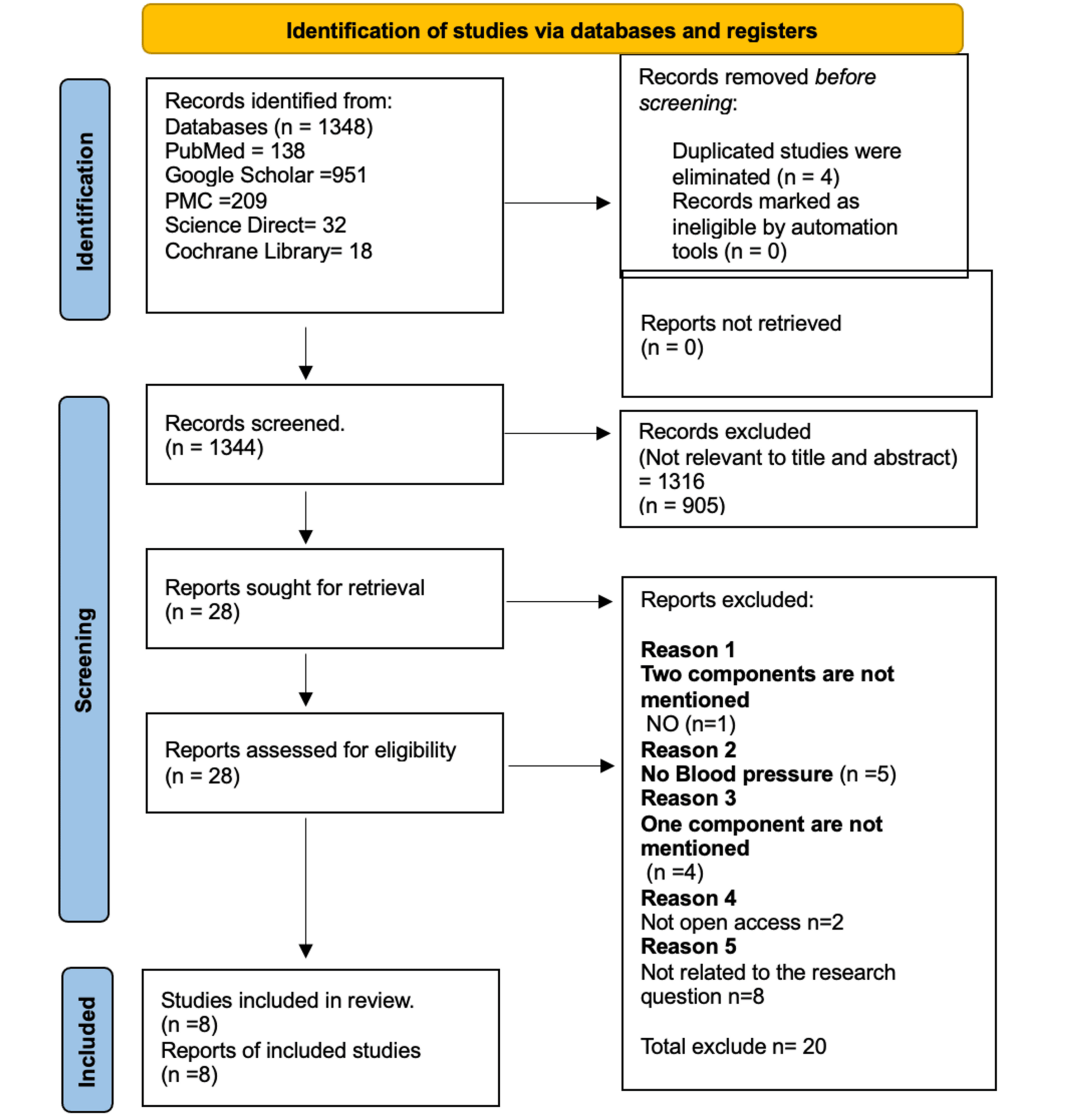
The PRISMA flowchart showing the article identification process. PRISMA: Preferred Reporting Items for Systematic Reviews and Meta-Analyses; PMC: PubMed Central.

Initially, the number of related papers identified on PubMed, PubMed Central, Google Scholar, Science Direct, and Cochrane Library was 1,348. No articles were deleted automatically from Google Scholar. Four duplicated papers were removed. We reviewed 1344 papers in detail and screened them by titles and abstracts. Also, we checked for the presence of the full-text. Therefore, 28 studies were selected for data extraction, and 1,316 studies were removed. The selected papers were assessed utilizing inclusion and exclusion criteria, while 20 papers were excluded. We thoroughly reviewed eight papers for quality, utilizing the corresponding quality assessment method. The PRISMA flowchart, which describes the identification and evaluation, is presented below in Figure 1.

For assessing the quality of non-randomized clinical trials, observational, pilot, and cross-sectional studies, we used the JBI tool [13]. We used the AMSTAR checklist for one systematic review study and used the Narrative Review Checklist for three narrative review studies. Risk of bias was evaluated by two authors independently, and a third reviewer checked the assessment results. Based on the suitable quality appraisal tools, the articles were organized based on their qualities as high, low, and medium. If a study scores greater than 70%, it is classified as high quality, studies with a score of 50-70% are classified as medium quality, and articles with a score of lower than 50% are classified as low quality.

The assessment criteria are demonstrated in Tables 2, 3. The quality appraisal of narrative review studies is summarized in Table 4, the systematic review assessment using the AMSTAR checklist is presented in Table 5, and a summary of all included studies is provided in Table 6.

**Table 2:** The Joanna Briggs Institute (JBI) quality appraisal tool is used for two non-randomized clinical trial studies and one pilot study. Based on the JBI quality appraisal tool questions, if a study obtains a “YES,” then it meets the assessment criteria. If the study obtains “NO,” it means the study does not meet the assessment criteria. If the information is not applicable, then it obtains N/A. If the information is not clear, the study obtains “?”.

JBI critical appraisal questions	Nordgren et al. (2012) [16]	Stavropoulos-Kalinoglou et al. (2013) [17]	Bartlett et al. (2018) [18]
1. Is it clear in the study what is the "cause" and what is the "effect" (i.e., there is no confusion about which variable comes first)?	Yes	Yes	Yes
2. Were the participants included in any comparisons similar?	No	Yes	Yes
3. Were the participants included in any comparisons receiving similar treatment/care, other than the exposure or intervention of interest?	No	Yes	No
4. Was there a control group?	No	Yes	No
5. Were there multiple measurements of the outcome, both pre- and post-intervention/exposure?	Unclear	Yes	Yes
6. Was follow-up complete, and if not, were differences between groups in terms of follow-up adequately described and analyzed?	N/A	Yes	Yes
7. Were the outcomes of participants included in any comparisons measured in the same way?	Yes	No	N/A
8. Were outcomes measured in a reliable way?	Yes	Yes	Yes
9. Was an appropriate statistical analysis used?	Yes	Yes	Yes
Overall appraisal	4/9 (44%)	8/9 (88.8%)	6/9 (66.6%)
Comments (Including reason for exclusion)	Excluded	Included	Excluded

**Table 3:** The Joanna Briggs Institute (JBI) tool for observational study. Based on the JBI quality appraisal tool questions, if a study obtains “YES,” it means the study meets the assessment criteria. If a study obtains “NO,” it means the study does not meet the assessment criteria. If the information is not applicable, then it obtains N/A. If the information is not clear, the study obtains “?”.

Questions	Metsios et al. (2009) [19]
1. Were the criteria for inclusion in the sample clearly defined?	Yes
2. Were the study subjects and the setting described in detail?	No
3. Was the exposure measured in a valid and reliable way?	Yes
4. Were objective, standard criteria used for measurement of the condition?	Yes
5. Were confounding factors identified?	No
6. Were strategies to deal with confounding factors stated?	No
7. Were the outcomes measured in a valid and reliable way?	Yes
8. Was an appropriate statistical analysis used?	Yes
Overall appraisal	5/8 (62.5%)

**Table 4:** The Narrative Review Checklist for three narrative review studies. According to the Narrative Review Checklist quality appraisal tool questions, if a study obtains “YES,” then the study meets the assessment criteria. If the study obtains “NO,” it means the study does not meet the assessment criteria. If a study obtains “?,” it means it is not clear whether the study meets or does not meet the assessment criteria.

Checklist item	Zegkos et al. (2016) [20]	Fedorchenko et al. 2025 [21]	Metsios et al. 2015 [22]
Identify the report as a narrative review	No	No	Yes
Provide an unstructured summary including: background, objective, a brief summary of the narrative review, and implications for future research, clinical practice, or policy development	Yes	Yes	Yes
Describe the rationale for the review in the context of what is already known	Yes	Yes	Yes
Specify the key question(s) identified for the review topic	Yes	Yes	Yes
Specify the process for identifying the literature search (e.g., years considered, language, publication status, study design, databases covered)	No	Yes	No
Discuss: (1) research reviewed, including fundamental/key findings, (2) limitations/quality of research reviewed, and (3) need for future research	Yes	Yes	Unclear
Provide overall interpretation of the review in the context of clinical practice, the Nutrition Care Process, policy development/implementation, or future research	Yes	Yes	Yes
Quality score	5/7 (71.4%)	6/7 (85.7%)	5/7 (71.4%)
Quality rating	Good	High	Good

**Table 5:** The AMSTAR checklist was used for the systematic review study. AMSTAR: Assessment of Multiple Systematic Reviews. Based on the AMSTAR checklist quality appraisal tool questions, the study is able to obtain "Yes" if the study meets the criteria of assessment, "No" if the study does not meet the criteria of assessment, and “Partial - Yes” if a study meets the assessment criteria in some points but not in total.

Item	Question	Metsios et al. (2008) [23]
1	Did the research questions and inclusion criteria include the components of PICO (Population, Intervention, Comparator, Outcome, Timeframe)?	Yes
2	Were review methods established prior to conducting the study, and were deviations justified?	Partial – Yes
3	Did the authors explain their selection of study designs for inclusion?	Yes
4	Was a comprehensive literature search strategy used?	Partial – Yes
5	Was study selection performed in duplicate?	No
6	Was data extraction performed in duplicate?	No
7	Did the authors provide a list of excluded studies and justify exclusions?	No
8	Were the included studies described in adequate detail?	Yes
9	Was the risk of bias in individual studies adequately assessed?	No
10	Were sources of funding for the included studies reported?	No
11	If a meta-analysis was performed, were appropriate methods used for the statistical combination of results?	N/A
12	If a meta-analysis was performed, did the authors assess the impact of risk of bias on the results?	N/A
13	Were methods for assessing publication bias (e.g., funnel plot, Egger’s test) described?	No
14	Was heterogeneity in results explained and discussed?	Yes
15	If quantitative synthesis was performed, was publication bias assessed and discussed?	No
16	Did the authors report potential conflicts of interest or funding sources?	Yes
Overall quality		7/16 (43.7%) – Low quality

**Table 6:** Summary of the included studies. RA: rheumatoid arthritis; CVD: cardiovascular disease; CRF: cardiorespiratory fitness; HDL: high-density lipoprotein; Vo2 max: aerobic capacity; HIIT: high-intensity interval training; RCT: randomized clinical trial.

Reference	Year of publication	Study design	Participants	Results/Key findings	Conclusion
Metsios et al. [22]	2015	Narrative review	N/A	Rheumatoid arthritis (RA) is an autoimmune condition affecting 0.5–1% of the general population. Signs and symptoms include articular and periarticular swelling, pain, and stiffness, which can cause significant mobility limitations. RA is strongly associated with cardiovascular disease (CVD) and other conditions such as lung disease, infections, and certain cancers. CVD-related mortality in RA patients is higher than in the general population. Chronic inflammation is the primary cause of CVD, while exercise effectively reverses inflammation and CVD risk factors.	RA is an autoimmune disease associated with CVD due to chronic inflammation. Physical activity plays a key role in reducing CVD risk in RA patients.
Fedorchenko et al. [21]	2025	Narrative review	345	RA in older adults presents complex clinical challenges due to age-related comorbidities and musculoskeletal degeneration, leading to reduced mobility and quality of life. This study explored rehabilitation interventions to improve physical activity (PA) in RA patients. Structured PA, including high-intensity walking, reduced blood pressure, heart rate, disease activity, pain, and fatigue.	RA in older adults is complicated by comorbidities and immobility. Structured PA is effective in lowering blood pressure, heart rate, and disease activity.
Zegkos et al. [20]	2016	Narrative review	N/A	The increased cardiovascular morbidity and mortality in RA patients are independent of traditional risk factors. The cumulative effect of inflammation and antirheumatic medications contributes to CVD development. Effective management requires coordination among cardiologists, rheumatologists, and exercise physiologists. Lifestyle modification and structured exercise are crucial for optimizing cardiovascular outcomes and alleviating symptoms such as pain and fatigue.	RA is strongly linked to CVD morbidity and mortality. Multidisciplinary care and lifestyle modification are essential to improve cardiovascular health.
Metsios et al. [19]	2009	Observational study	65	RA patients exhibit reduced physical activity levels and increased CVD-related morbidity and mortality. Participants were categorized by activity level: active, moderately active, or inactive. Those who were highly active demonstrated significant improvements in systolic blood pressure and low-density lipoprotein (LDL) cholesterol.	RA patients experience higher CVD risk, but physical activity significantly improves cardiovascular health and overall outcomes.
Metsios et al. [23]	2008	Systematic review	–	The incidence and prevalence of CVD in RA patients are elevated, but exercise is recognized as a vital preventive strategy. Physiological adaptations from exercise can protect against CVD even with existing risk factors. However, there is debate regarding optimal exercise intensity and duration. Despite fear of disease aggravation, physical activity is now proven to improve overall health and slow joint damage without worsening RA.	CVD prevalence is high in RA patients. Exercise is one of the most effective methods for CVD prevention and health improvement in this population.
Stavropoulos-Kalinoglou et al. [17]	2013	Non-RCT	N/A	Poor cardiorespiratory fitness (CRF) is a strong predictor of CVD. In this non-randomized study, 40 participants were divided into exercise and control groups. Baseline characteristics were similar, but post-intervention results showed improvements in blood pressure, high-density lipoprotein (HDL) levels, and VO₂ max in the exercise group.	Low CRF increases CVD risk in RA patients. Physical activity significantly improves CRF and related cardiovascular parameters.
Nordgren et al. [16]	2012	Non-RCT	900	People with RA experience disability and premature mortality, primarily due to CVD linked to physical inactivity. The study showed that regular exercise reduces pain, improves function, and lowers CVD risk in RA patients.	Physical inactivity contributes to CVD and disability in RA patients. Exercise improves pain, mobility, and cardiovascular outcomes.
Bartlett et al. [18]	2018	Pilot study	N/A	RA is a chronic inflammatory condition that causes joint damage and discourages physical activity, increasing susceptibility to infections. This 10-week high-intensity interval training (HIIT) walking program for 12 sedentary RA patients resulted in improved cardiorespiratory fitness, aerobic capacity, blood pressure, and heart rate.	RA is an inflammatory autoimmune disease associated with CVD. Structured physical activity, particularly HIIT, improves cardiovascular and overall health in RA patients.

Study Features

After the selection and quality appraisal process, we included eight related papers. Three articles are narrative reviews [20-22], two articles are non-randomized clinical trials [16,17], one study is observational [19], one paper is a pilot study [18], and one article is a systematic review [23].

Discussion

This review highlights the consistent association between RA and increased CVD risk, and the emerging role of physical activity (PA) in potentially mitigating this burden. The eight included studies [16-23] suggest that structured PA may improve cardiovascular outcomes, reduce disease activity, and enhance quality of life in RA patients.

Synthesis of Findings

Narrative reviews [20-22] emphasized systemic inflammation as a central driver of cardiovascular risk in RA. They noted that structured exercise interventions can help reduce this burden by improving blood pressure, lipid levels, and symptoms such as fatigue and stiffness. The systematic review by Metsios et al. (2008) [23] supported these observations but acknowledged uncertainty regarding the most effective exercise intensity and duration. Observational data [19] indicated that higher levels of PA were associated with more favorable cardiovascular profiles. Interventional studies reported tangible clinical benefits. Nordgren et al. (2012) [16] and Stavropoulos-Kalinoglou et al. (2013) [17] described reduced CVD risk and improved cardiorespiratory fitness. Bartlett et al. (2018) [18] found that high-intensity interval training enhanced aerobic capacity and immune function in older adults with RA.

Comparison With Broader Literature

Beyond our included studies, additional literature supports these findings. Metsios et al. (2014) [24] showed that individualized exercise programs can improve endothelial function in RA patients, reinforcing the vascular benefits of PA. A systematic review by Tierney et al. (2012) [25] confirmed the general safety and effectiveness of PA interventions, while Munneke et al. (2005) [26] demonstrated that high-intensity, weight-bearing exercise does not accelerate radiologic joint damage. Furthermore, Cochrane evidence [27] supports the use of dynamic exercise therapy as a safe and effective adjunct to RA management. Taken together, these studies strengthen the view that PA is both secure and clinically valuable in RA.

Critical Appraisal and Limitations

This review, conducted in accordance with PRISMA 2020 guidelines [12], synthesized evidence from non-randomized clinical trials, cross-sectional studies, and one systematic review. While these studies provided important insights, their modest sample sizes, short follow-up periods, and methodological heterogeneity limit the strength of causal inferences. Patient concerns about worsening symptoms, together with comorbidities and reduced mobility, continue to restrict participation in structured PA programs, highlighting challenges in translating evidence into practice. Regarding limitations, only English-language articles were included, potentially excluding relevant studies. No time filter was applied due to the limited availability of eligible papers, potentially introducing variability across study periods. Additionally, one study could not be fully assessed because the full text was unavailable.

Future Research

Based on this systematic review, numerous studies have investigated the effects of exercise on cardiovascular risk factors in RA; however, few have specifically examined its direct impact on blood pressure, an area that warrants further research. In addition, future research should include long-term RCTs to evaluate adherence to exercise programs, their sustained cardiovascular benefits, and their effect on CVD mortality in RA. Education regarding the cardiovascular aspects of RA and the role of exercise, directed at both patients and healthcare professionals, remains essential to support lasting lifestyle changes through multidisciplinary approaches.

## Conclusions

Based on this systematic review, the available evidence suggests that the most critical factors in improving CVD and CV risk factors in RA patients are lifestyle modifications (structured exercise and stress reduction), control of disease activity with disease-modifying anti-rheumatic drugs, and medications for controlling traditional risk factors, such as antihypertensive agents. Many studies recommend a multidisciplinary approach involving exercise physiologists, cardiologists, and other specialists for the management of RA patients. Still, the main cornerstone of the treatment is exercise at any stage of the disease, since it improves patients' general health, including CVD and CV risk factors like blood pressure, and reduces physical immobility.
